# Re-Discovery of Pyrimidine Salvage as Target in Cancer Therapy

**DOI:** 10.3390/cells11040739

**Published:** 2022-02-20

**Authors:** Melanie Walter, Patrick Herr

**Affiliations:** Weston Park Cancer Centre, Department of Oncology and Metabolism, University of Sheffield, Sheffield S10 2RX, UK; mwalter3@sheffield.ac.uk

**Keywords:** nucleotide metabolism, cancer therapy, DNA replication, replication stress, pyrimidine salvage

## Abstract

Nucleotides are synthesized through two distinct pathways: de novo synthesis and nucleoside salvage. Whereas the de novo pathway synthesizes nucleotides from amino acids and glucose, the salvage pathway recovers nucleosides or bases formed during DNA or RNA degradation. In contrast to high proliferating non-malignant cells, which are highly dependent on the de novo synthesis, cancer cells can switch to the nucleoside salvage pathways to maintain efficient DNA replication. Pyrimidine de novo synthesis remains the target of interest in cancer therapy and several inhibitors showed promising results in cancer cells and in vivo models. In the 1980s and 1990s, poor responses were however observed in clinical trials with several of the currently existing pyrimidine synthesis inhibitors. To overcome the observed limitations in clinical trials, targeting pyrimidine salvage alone or in combination with pyrimidine de novo inhibitors was suggested. Even though this approach showed initially promising results, it received fresh attention only recently. Here we discuss the re-discovery of targeting pyrimidine salvage pathways for DNA replication alone or in combination with inhibitors of pyrimidine de novo synthesis to overcome limitations of commonly used antimetabolites in various preclinical cancer models and clinical trials. We also highlight newly emerged targets in pyrimidine synthesis as well as pyrimidine salvage as a promising target in immunotherapy.

## 1. Introduction

The essential building blocks of DNA, as well as RNA, consist of two classes of nucleotides, purines, and pyrimidines. Both nucleotides are composed of nucleobases such as the purine precursors adenine (A) and guanine (G), as well as the pyrimidine nucleobases thymine (T), cytosine (C), and uracil (U), respectively. These nucleobases are converted to nucleosides when linked to either ribose or deoxyribose, and nucleotides with the further addition of one to three phosphate groups to the purine or pyrimidine moiety.

Nucleotides are synthesized via two distinct pathways: the de novo synthesis, which utilizes amino acids and glucose, and the salvage pathway. The de novo biosynthesis of nucleotides is a highly energy-intensive multistep process using six to ten molecules of ATP per generated nucleotide and is the main source for nucleotide synthesis in non-malignant cells [1]. A multitude of dedicated enzymes regulates not only the generation of nucleosides but also maintains a fine balance in nucleotide pool composition through allosteric inhibitory mechanisms [2]. To maintain high proliferation, cancer cells can switch to the more energy-efficient nucleoside salvage pathways [1,3]. Whereas the role of purine salvage has been reviewed previously, the significance of pyrimidine salvage in cancer therapy has yet to be fully established [4,5,6].

With the discovery of pyrimidine de novo synthesis as an attractive target in cancer therapy more than two decades ago, various anti-cancer agents and pyrimidine analogs were developed and are still used in cancer therapy to date [7,8,9,10]. However, cancer cells can escape pyrimidine de novo synthesis inhibition by adapting the nucleoside salvage pathways leading to unsuccessful market approval of novel compounds as well as limitations of currently used anti-cancer agents [11,12,13].

Here we focus on pyrimidine synthesis in cancer therapy and discuss the recent re-discovery of targeting pyrimidine salvage to overcome observed limitations of currently used anti-cancer agents and pyrimidine analogs. Furthermore, we highlight co-targeting of pyrimidine de novo synthesis and salvage pathways as a novel strategy in cancer therapy.

## 2. Pyrimidine De Novo and Salvage Pathways

**Figure 1:**
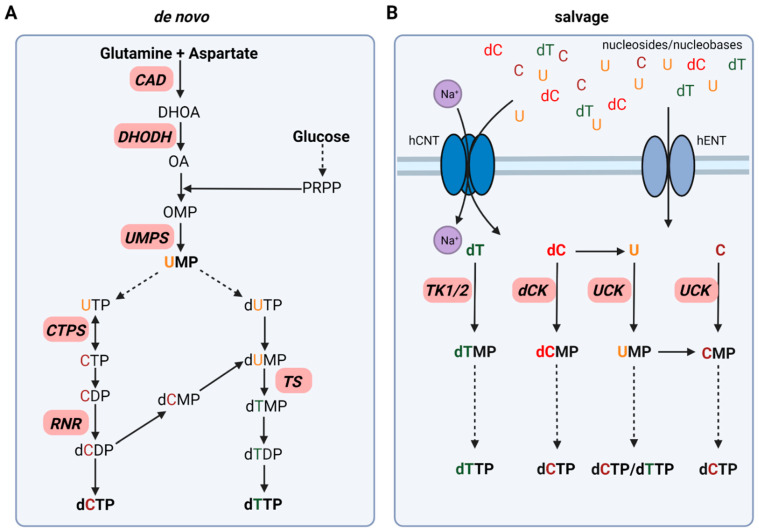
Simplified schematic of pyrimidine synthesis divided into de novo synthesis (**A**) and salvage pathways (**B**). Enzymes of interest for targeting approaches in cancer therapy are displayed in red. Solid arrows display direct steps in pyrimidine synthesis. Dashed arrows represent multiple steps leading to the synthesis of the corresponding pyrimidine. Created with BioRender.com (accessed on 20 January 2022).

In mammalian cells, pyrimidines are derived through de novo synthesis as well as salvage pathways (Figure 1) [14,15]. Pyrimidine synthesis in healthy non-malignant fast proliferating cells relies predominantly on the de novo biosynthesis to maintain the demand of pyrimidines for successful DNA replication. In contrast, differentiated non-malignant cells use predominantly salvage pathways for the maintenance of pyrimidine synthesis [1,15].

Cancer cells have however frequently undergone metabolic rewiring to exploit the more energy-efficient pyrimidine salvage pathway to maintain faithful DNA replication in highly proliferating cells and, consequently, support genome integrity [1,15].

### 2.1. Pyrimdine De Novo Synthesis

Pyrimidine de novo synthesis requires glucose and the two amino acids glutamine and aspartate as starting points for the synthesis of both, deoxythymidine triphosphate (dTTP) and deoxycytidine triphosphate (dCTP). In the first committed step of pyrimidine synthesis, the trifunctional enzyme CAD converts glutamine and aspartate to *N*-carbamoyl-aspartate and, dihydroorotate (DHOA) resulting in a pyrimidine ring formation. The mitochondrial membrane protein dihydroorotate dehydrogenase (DHODH) catalyzes the formation of orotate (OA), which is then transformed into orotidine monophosphate (OMP) upon addition of 5-phosphoribosyl-1-phosphate (PRPP). OMP is further metabolized to the main pyrimidine precursor uridine monophosphate (UMP) by UMP synthase (UMPS) (Figure 1A) [14].

For dCTP synthesis, UMP is phosphorylated to uridine triphosphate (UTP) via cytidine monophosphate kinase (CMPK) and nucleoside diphosphate kinase (NDPK) followed by the formation of CTP by the bidirectional CTP synthase (CTPS). After dephosphorylation of CTP to CDP by NDPK, CDP is further reduced to deoxycytidine diphosphate (dCDP) by ribonucleotide reductase (RNR). NDPK then catalyzes the formation of dCTP, which can then be incorporated in DNA (Figure 1A) [14].

In contrast to dCTP synthesis directly via RNR, dTTP synthesis is dependent on the formation of deoxythymidine diphosphate (dTDP) via deoxyuridine monophosphate (dUMP) formation. dUMP can be synthesized upon deoxyuridine triphosphate (dUTP) generation catalyzed by dUTPase, which is then dephosphorylated to dUMP. In addition, dUMP formation occurs upon the switch from dCMP to dUMP by deoxycytidylate deaminase (DCTD). Thymidylate synthase (TS), as well as deoxythymidine monophosphate (dTMP) kinase, are required to form dTMP and dTDP. NDPK phosphorylates dTDP to dTTP for DNA incorporation (Figure 1A) [14].

### 2.2. Pyrimidine Salvage Pathways

Pyrimidine salvage utilizes extracellular nucleosides and nucleobases via uptake from the bloodstream or intracellular recycled nucleic acids (UMP, CMP, TMP) derived from DNA and RNA degradation, to synthesize nucleotides for efficient DNA replication and repair as well as mRNA synthesis. Two different types of nucleoside transporter families have been identified: the Na^+^-dependent SLC28 family of concentrative nucleoside transporter (CNT) and the Na^+^-independent SLC29 family equilibrative nucleoside transporter (ENT) (Figure 1B) [16].

After cellular uptake, free pyrimidines are converted to their corresponding nucleoside and deoxynucleoside monophosphates (NMPs/dNMPs). Two enzyme classes are responsible for this process: deoxycytidine kinase (dCK) as well as the thymidine kinases (TKs) cytosolic thymidine kinase 1 (TK1) and mitochondrial thymidine kinase 2 (TK2). NMPs are then further phosphorylated to their corresponding deoxynucleoside triphosphates (dNTPs) as discussed above. Deoxycytidine (dC) can be converted to uracil (U) by cytidine deaminase (CDA), which is then further phosphorylated to UMP by UCK. In addition, this switch from C to U can also take place at the monophosphate level. DCTD, as mentioned previously, catalyzes the formation of UMP from CMP and, therefore, contributes to pyrimidine salvage (Figure 1B) [14].

## 3. Limitations of Targeting Pyrimidine De Novo Synthesis in Cancer

Pyrimidine synthesis and, more specifically, targeting the de novo pyrimidine synthesis pathways remains the backbone of cancer therapy for several decades. The hitherto most prominent group of anti-cancer agents are the so-called nucleoside analogs/anti-metabolites with 5-fluorouracil (5-FU), gemcitabine, and cytarabine as the most prominent pyrimidine analogs (Table 1) [17,18,19,20]. Nucleoside analogs are structurally similar to their physiological nucleoside counterparts and exhibit their mode of action either through incorporation into DNA or RNA or via inhibition of enzymes involved in the nucleotide de novo synthesis pathways.

The inhibition of CAD to impair pyrimidine de novo synthesis in the first committed step from glutamine was thought to be a promising strategy in cancer already in the early 1970s (Figure 1A). One of the most studied CAD inhibitors is *N*-(phosphonacetyl)-*L*-aspartate (PALA), which initially showed beneficial effects in vitro but failed in clinical studies later on (Table 2) [21,22,23].

In contrast to CAD inhibitors, several DHODH inhibitors including brequinar (BRQ) and teriflunomide as well as its prodrug leflunomide have reached market approval as immunosuppressive agents in rheumatoid arthritis and multiple sclerosis. As dihydroorotate dehydrogenase (DHODH) converts dihydroorotate to orotate in UMP de novo synthesis and antitumor properties were observed in several cancer tissues, the focus shifted towards DHODH as a target in cancer therapy (Figure 1A) [9,11,24]. Preliminary studies in vitro and in vivo showed promising results. However, the observed antitumor activity, as well as tumor growth inhibitory effects, could not be reproduced in Phase II clinical trials (Table 2) [25,26,27,28,29]. In recent years, multiple studies in different cancer cell and animal models, as well as patient-derived cancer cells and xenograft models, once again elucidated the importance of targeting DHODH alone or in combination with other anti-cancer agents. This renewed interest in DHODH has led to the development of new inhibitors as well as the re-discovery of BRQ and related agents. However, none of the novel nor already developed inhibitors has gained market approval for anti-cancer therapy so far [30,31,32].

Pyrazofurin is a nucleoside analog that inhibits the orotidine monophosphate decarboxylase function of UMPS and showed initially promising results in in vitro studies in several cancer cells lines (Figure 1A). Nevertheless, in the late 1970s, it has failed to proceed beyond Phase II clinical trials in several cancers due to lack of efficacy and severe toxicity (Table 2) [33,34,35,36,37].

Targeting thymidine synthase (TS) with 5-FU or its prodrug capecitabine remains the backbone of anti-cancer therapy with its greatest impact in the prolongation of overall survival in advanced colorectal cancer (Table 1; Figure 1A) [20,38]. After uptake into the cell, 5-FU is metabolized to its active metabolites fluorodeoxyuridine monophosphate (FdUMP), and fluorouridine triphosphate (FUTP). Whereas FUTP impairs RNA synthesis via its incorporation into mRNA, FdUMP covalently inhibits TS resulting in pyrimidine synthesis disruption and cancer cell death [20]. Even though 5-FU is still widely used in clinical practice, it comes with certain limitations such as low response rates as well as resistance in cancer patients [13,20].

One of the main reasons why initial in vitro findings of most inhibitors of the pyrimidine de novo synthesis could not be translated in clinical studies and the observed low response rates of cancer patients is the ability of cancer cells to exploit the more energy-efficient nucleoside salvage pathway to escape pyrimidine de novo synthesis inhibition [2,9,11,39,40,41]. Pyrimidine salvage utilizes free nucleosides present in the extracellular tumor environment to maintain efficient DNA replication and cell proliferation. Uridine concentrations in human plasma and serum range from 5–20 μM, which makes it the most dominant circulatory pyrimidine when compared to plasma levels of the other two pyrimidines cytidine and thymidine with 0.6 μM and 0.2 μM, respectively [42]. Uridine is not only the most prominent circulatory pyrimidine but also the most prominent nucleoside when compared with physiological purine plasma levels of approximately 0.5 μM for adenosine and 0.9 μM for guanosine [43,44]. This highlights the need for novel strategies targeting the pyrimidine salvage pathways.

## 4. Pyrimidine Salvage as Target in Cancer Therapy

Previous strategies to exploit pyrimidine de novo synthesis inhibition suffered mostly from the unsuccessful translation of in vitro and in vivo findings to clinical trials. The cell’s ability to shift to pyrimidine salvage to maintain DNA replication and cell proliferation opened up a new field of novel targets in pyrimidine synthesis.

### 4.1. Nucleoside Transporter

Nucleoside transporters (NTs) are transmembrane proteins for the import and export of free nucleosides and nucleobases from the extracellular environment of cancer and non-cancer cells and, thus, are involved in nucleoside salvage (Figure 1B). NTs are members of the solute carrier protein family and are classified in two structural unrelated NT families; the human concentrative transporter (hCNT; SLC28) and the human equilibrative transporter family (hENT, SLC29). Substrate specificity, uptake efficiency, expression levels, and location of NTs vary between the different transporter families as well as between family members. Whereas hCNTs are Na^+^-dependent unidirectional nucleoside import pumps that transport nucleosides against their concentration gradients, hENTs function as bidirectional Na^+^-independent NTs [45,46,47,48,49,50].

Even though all three members of the CNT family transport uridine as well as both, hCNT1 and hCNT3 transport all pyrimidines; recent studies suggest the role of hCNTs as transceptors in nucleoside sensing and signal transduction instead of nucleoside homeostasis [51]. Together with the observed decrease or loss in hCNT1 expression in different tumors and the lack of currently developed hCNT inhibitors, nucleoside uptake and thus nucleoside homeostasis via hENTs remains the target of interest to inhibit pyrimidine uptake and therefore salvage in anti-cancer therapy [52,53]. Out of the four hENT family members, only hENT1 and hENT2 are widely expressed at cell plasma membranes of various tissues and both are required for pyrimidine transport [54]. Furthermore, hENT2 was identified as a key element to maintain the supply of nucleosides and nucleotides for DNA replication and cell cycle progression [55].

**Figure 2:**
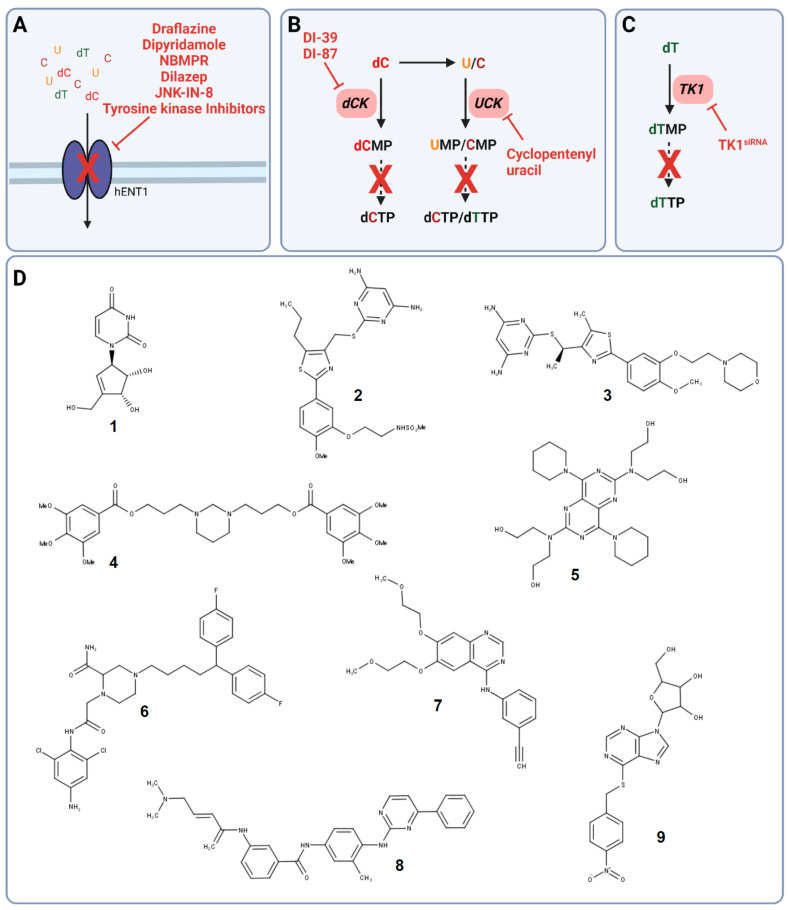
Targets in pyrimidine salvage and their corresponding inhibitors. (**A**) Inhibition of pyrimidine uptake transporter hENT1. (**B**) Targeting of either dCK with DI-39 and DI-87 or UCK with cyclopentenyl uracil. (**C**) Silencing of *TK1* with TK1siRNA leads to dTTP synthesis inhibition. (**D**) Chemical structures of cyclopentenyl uracil (**1**), DI-39 (**2**), DI-87 (**3**), the tyrosine kinase inhibitor erlotinib (**4**), dilazep (**5**), dipyridamole (**6**), draflazine (**7**), JNK-IN-8 (**8**), and nitrobenzylmercaptopurine riboside (NBMPR) (**9**). Solid arrows are direct steps and dashed arrows represent multiple steps in pyrimidine salvage. Created with BioRender.com (accessed on 20 January 2022).

The two hENT transporters can be differentiated by their activity towards the nucleoside analog nitrobenzylmercaptopurine riboside (NBMPR), a potent hENT1 inhibitor and nucleoside analog (Figure 2A,D) [49].

Already in the 1980s and 1990s, the vasodilators dipyridamole and dilazep were identified to inhibit nucleoside transport via targeting hENT1 and, however less potent, hENT2 (Figure 2A,D) [56,57,58]. Even though targeting nucleoside uptake in combination with other cytotoxic agents was thought to be a promising anti-cancer strategy, clinical phase I studies did not show the desired efficacy, and targeting nucleoside uptake moved out of the focus [59,60,61,62,63,64]. Only recently, the potential of dipyridamole to reduce triple-negative breast cancer progression and metastasis in xenograft models was uncovered, which has to be further evaluated in clinical trials [65].

The failure of dipyridamole in the clinics can be explained by its observed binding to serum protein α_1_-acid glycoprotein (AGP) causing insufficient target engagement and, therefore, the low response rate in vivo as well as in cancer patients [63,64]. To overcome the observed limitations, new chemically optimized hENT1 and hENT2 inhibitors were developed and identified. Structural analogs of the platelet aggregation and hENT1 inhibitor draflazine were developed to prolong the drug resiliency time leading to improved binding affinity as well as kinetic properties compared to dipyridamole and dilazep (Figure 2A,D) [66]. Furthermore, screens to assess off-target effects of tyrosine kinase inhibitors revealed the potential of several tyrosine kinase inhibitors such as lorlatinib, gefitinib, vandetanib, and erlotinib to not just inhibit their designated target but also hENT1 causing nucleotide transport inhibition in non-cancer and cancer cells (Figure 2A,D) [67,68,69]. In addition, hENT1 inhibition and, thus, impaired nucleoside uptake was observed upon treatment with the C-Jun N-terminal kinase (JNK) inhibitor JNK-IN-8 in pancreatic cancer cells demonstrating another potential drug class to target pyrimidine salvage in cancer (Figure 2A,D) [70].

### 4.2. Uridine-Cytidine Kinase and Deoxycytidine Kinase

After uptake of free uridine from the extracellular tumor environment, UCK phosphorylates uridine to UMP, the main precursor for dUTP, dCTP, and dTTP (Figure 1B). Whereas UCK is also required for the direct phosphorylation of cytidine, dCK phosphorylates deoxycytidine, representing another way to synthesize dCTP for DNA synthesis and replication (Figure 1B).

Uridine has been shown to have a significant role in countering pyrimidine de novo inhibition by several anti-cancer agents, leading to unsuccessful clinical trial outcomes. Already in the mid-1980s and early 1990s, targeting of UCK by small molecule inhibitors was proposed as a novel strategy in several cancers. Cyclopentenyl uracil was identified as a selective inhibitor for UCK, reducing the salvage of uridine and to lesser extent cytidine, making it an interesting candidate for use as chemotherapeutic (Figure 2B,D) [12,71].

However, even though cyclopentenyl uracil and other UCK and dCK inhibitors were identified and their potential use as anti-cancer agents was proposed, this approach was not followed up until recently with the discovery of the link between dCK and replication stress in acute lymphoblastic leukemia (ALL). The knockout of dCK and, therefore, impaired pyrimidine salvage in mouse models of hematological cancer, induced replication stress followed by S phase arrest and DNA damage in hematopoietic progenitors due to a decreased dCTP pool [2]. This observation resulted in the development of the small molecule dCK inhibitor DI-39, which induced replication stress in ALL cancer cell models through dCTP depletion (Figure 2B,D) [72].

Even though DI-39 showed promising results as a single agent and more prominently as combination therapy with other inhibitors of pyrimidine de novo synthesis in ALL cancer cells and mouse models, DI-39 has limited solubility and metabolic stability due to a short half-life in vivo leading to the development of additional dCK inhibitors with DI-87 being the most promising candidate (Figure 2B,D) [73,74]. The newly developed small molecule DI-87 showed promising pharmacological effects in vitro as well as in vivo ALL models [73].

### 4.3. Thymidine Kinases as a Prognostic Biomarker and Anti-Cancer Target

Thymidine kinases (TKs) convert free thymidine after its uptake from the extracellular matrix into thymidine monophosphate, which is then further phosphorylated and incorporated into the DNA (Figure 1B). There are two thymidine kinase genes in humans, encoding for the cytosolic cell-cycle dependent TK1 and the mitochondrial TK2. *TK2* is continuously expressed in low amounts during the cell cycle whereas *TK1* expression and abundance are increased in the S/G2 phase in proliferating cells [75,76]. *TK1* expression is upregulated during the early stages of cancer development and elevated levels are detected in the serum of cancer patients making it an ideal biomarker [77,78,79,80]. Several studies showed that high expression of *TK1* correlates with poor prognosis, reduced overall survival, and relapse in patients with lung, breast, or pancreatic cancer [81,82,83].

Silencing of *TK1* decreased cell proliferation in vitro and in vivo in pancreatic ductal adenocarcinoma (PDAC) cell lines suggesting the exploitation of TK1 not just as a biomarker but also as a potential anti-cancer target (Figure 2C) [82]. In addition, *TK1* silencing in thyroid carcinoma cell lines caused a decrease in cell proliferation, invasion, and migration and induced apoptosis. These findings were supported by inhibition of tumor growth in thyroid carcinoma xenograft studies, further highlighting the role of TK1 in cancer [84]. Strikingly, there are no TK1 inhibitors for the use in cancer described in the literature so far.

With the recent discovery that TK1 localizes to the plasma membrane of malignant cells only, TK1 is now also considered a potential anti-cancer target suitable for immunotargeting [85,86]. Consequently, the effects of monoclonal antibodies targeting TK1 were evaluated in lung, breast, colon, and prostate cancer cell models. The binding of TK1 monoclonal antibodies to their corresponding TK1 epitopes was observed in all cancer cell models but not in normal lymphocytes suggesting the suitability of anti-TK1 antibodies as a highly specific targeting approach in malignant cells. Furthermore, monoclonal antibodies could potentially be exploited to detect TK1 on tumor cells, and, therefore, determine tumor burden in cancer patients in a diagnostic approach. Furthermore, anti-TK1 antibodies induced cytolysis of lung and breast cancer cells by effector cells demonstrating the potential to be used as immunotargeting agents to eliminate high TK1 expressing tumor cells in cancer therapy [87]. However, this approach has not been evaluated in animal models so further studies are required to determine the translational aspect of targeting TK1 with monoclonal antibodies in cancer.

In contrast to TK1, the mitochondrial thymidine kinase TK2 has lower substrate specificity. In addition to phosphorylating thymidine, TK2 can also phosphorylate deoxycytidine to dCMP the precursor for dCTP [88,89]. The deoxycytidine analog gemcitabine (2′,2′-difluoro-2′-deoxycytidine; dFdC) is activated through the activity of another pyrimidine salvage pathway enzyme dCK via conversion to the monophosphate required for active gemcitabine metabolite formation [90]. However, a high level of dCTP leads to decreased cytotoxicity and anticancer activity of gemcitabine due to the negative feedback regulation of dCK activity [91]. Diminishing dCTP synthesis via TK2 siRNA knockdown caused an increase in anti-proliferative activity of gemcitabine upon an increase in dCK levels in cervical carcinoma as well as breast cancer cell models in vitro. This effect was not observed upon the siRNA-induced knockdown of the pyrimidine de novo synthesis enzyme TS suggesting not just a potential role of TK2 as an anti-cancer target but also its specific role in gemcitabine resistance [92].

## 5. Co-Targeting of Pyrimidine De Novo Synthesis and Salvage Pathways to Overcome Limitations of De Novo Synthesis Inhibitors

Uncovering the impact of pyrimidine salvage in the rescue of pyrimidine de novo synthesis inhibition as well as its potential as an anti-cancer target resulted in the rationalization of new strategies to co-target pyrimidine de novo synthesis and salvage pathways to overcome the limitations of targeting pyrimidine de novo inhibition alone already in the 1980s and 1990s [11,12]. Simultaneous inhibition of uridine salvage with cyclopentenyl uracil and pyrimidine de novo synthesis with the CAD inhibitor PALA increased cancer cell death in mouse models further highlighting the impact of nucleoside salvage on the efficacy of anti-cancer agents targeting de novo synthesis. Co-targeting pyrimidine salvage and de novo synthesis were therefore suggested to be beneficial in anti-cancer therapy [12]. As an example, co-targeting of DHODH with BRQ and nucleoside transport with dipyridamole increased the efficiency of DHODH in vitro and in vivo [11].

Even though preliminary results in vitro and in vivo demonstrated synergy of pyrimidine de novo synthesis and salvage inhibition leading to a beneficial response compared to pyrimidine de novo synthesis inhibition alone, this strategy was not followed up until recently.

### 5.1. Co-Targeting De Novo Pyrimidine Synthesis and Nucleoside Uptake

Even though anti-cancer agents targeting pyrimidine de novo synthesis via DHODH inhibition failed to prove their effectiveness in clinical trials, with advancing technologies and methodologies such as gene expression profiling and metabolomics, the importance of DHODH as a target in cancer was rediscovered. Consequently, an old approach to overcome the observed adaptations towards nucleoside salvage in cancer cells to escape growth inhibition was once again investigated [11].

**Figure 3:**
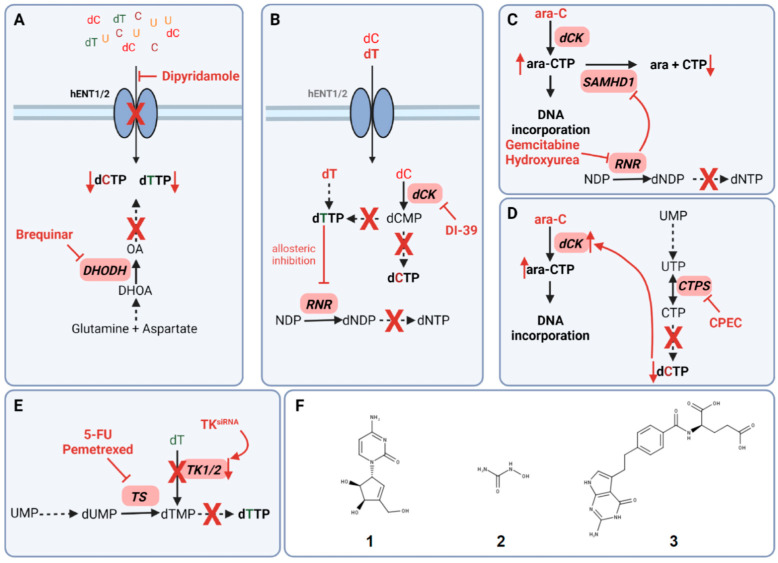
Principles of co-targeting pyrimidine de novo synthesis and salvage pathways. (**A**) Simultaneous targeting of pyrimidine uptake with dipyridamole and DHODH with brequinar. (**B**) Targeting of both, RNR via allosteric inhibition with TTP synthesized via dT addition, and dCK with DI-39. (**C**) RNR inhibition with gemcitabine or hydroxyurea results in non-allosteric inhibition of the ara-CTPase SAMHD1 leading to an increase in ara-CTP DNA incorporation. (**D**) CTPS inhibition with cyclopentenyl cytosine (CPEC) leads to an increase in dCK activity followed by increased ara-CTP DNA incorporation. (**E**) dTTP synthesis inhibition via co-targeting of TS with 5-fluorouracil (5-FU) or pemetrexed and siRNA knockdown of TK1/2. (**F**) Chemical structures of CPEC (**1**), hydroxyurea (**2**), and pemetrexed (**3**). Solid arrows represent direct steps in the pathway. Dashed arrows display multiple steps leading to metabolite synthesis. Created with BioRender.com (accessed on 20 January 2022).

Several studies proposed co-targeting DHODH with BRQ and nucleoside uptake via hENT1/2 with dipyridamole in different cancer cell models (Figure 3A). Synergistic effects were observed in colon cancer and pancreatic cancer cells [40,93,94]. However, the in vitro findings in colon cancer and pancreatic cancer cells could not be translated in in vivo xenograft cancer models due to no significant differences in tumor sizes after co-treatment with BRQ and dipyridamole compared to BRQ alone [40].

DHODH was identified to be an effective target in *MYCN*-amplified neuroblastoma cell lines and mouse neuroblastoma models. However, in contrast to the combination of BRQ and dipyridamole, DHODH inhibition with BRQ did not cause the suppression of proliferation and tumorigenicity of neuroblastoma cell lines when subjected to physiological uridine levels demonstrating again the need for co-targeting pyrimidine salvage. In addition, neuroblastoma growth was suppressed in animal models when subjected to co-treatment with BRQ and dipyridamole [94].

Synergistic effects of DHODH and hENT1/2 inhibition were also observed in acute myeloid leukemia (AML). Whereas the newly developed DHODH inhibitor MEDS433 had limited efficacy in vitro when subjected to physiological concentrations of uridine, combining DHODH inhibition and dipyridamole caused an increase in toxicity and, therefore, cell death in AML cells but not in non-cancer cells. High apoptotic rates were also observed in patient-derived primary AML cells suggesting the suitability of co-targeting DHODH and hENT1/2 in AML, which has to be further confirmed in vivo [93].

However, when combining pyrimidine de novo synthesis inhibitors with inhibitors of nucleoside uptake, the choice of de novo inhibitor is crucial. NTs and, more specifically, hENT1/2 are not only required for the uptake of free nucleosides and nucleobases but also the uptake of nucleoside analogs [53,95,96]. Consequently, nucleoside analogs such as gemcitabine that are depending on the uptake via hENT1/2 should not be used for combination therapy with dipyridamole or related compounds to maintain their activity and, thus efficacy in cancer therapy [95].

### 5.2. Co-Targeting of Ribonucleotide Reductase and Deoxycytidine Kinase

In pyrimidine de novo synthesis, ribonucleotide reductase is responsible for dCDP synthesis, which is then further converted to dCTP for DNA and RNA synthesis (Figure 1A). RNR activity can be impaired by either direct targeting with anti-cancer agents such as hydroxyurea or gemcitabine or via allosteric regulation upon dTTP levels (Figure 3B) [2,97]. More specifically, upon a high concentration of dTTP, RNR activity is inhibited through binding of dTTP to its regulatory site disabling CDP binding and, thus, interrupting dCTP de novo synthesis [2]. This regulation of RNR was exploited as a strategy in cancer therapy through the treatment with dT as a single dCTP-depleting agent via dTTP synthesis by TK1. However, clinical trials showed only limited efficacy due to the ability of cancer cells to exploit pyrimidine salvage for successful dCTP synthesis [2,98,99,100].

The ability of cancer cells to switch from pyrimidine de novo synthesis via RNR to pyrimidine salvage, to maintain efficient DNA synthesis, and to escape allosteric RNR inhibition with dT resulted in the development of a strategy to co-target RNR and the pyrimidine salvage enzyme dCK.

Simultaneous targeting of dCK with the small molecule inhibitor DI-39 and RNR with dT in acute lymphoblastic leukemia (ALL) cancer cells induced replication stress and apoptosis confirming synergy between de novo dCTP inhibition and pyrimidine salvage inhibition (Figure 2B). These findings were successfully translated to ALL in vivo models, where co-treatment with DI-39 and dT caused a decrease in tumor size with limited host toxicity [72].

These findings could also be replicated in glioblastoma cell lines. However, not all glioblastoma cell lines were sensitive towards simultaneous de novo pyrimidine synthesis and pyrimidine salvage inhibition highlighting the need for personalized treatment strategies for glioblastoma cancer patients. The effects of targeting dCTP de novo synthesis with DI-39 and salvage with dT could not be assessed in vivo due to poor blood–brain barrier penetration of both anti-cancer agents. To further investigate targeting both pyrimidine synthesis pathways in glioblastoma, alternatives for DI-39 and dT with good blood–brain barrier penetration abilities will have to be developed [101].

### 5.3. Co-Targeting of SAMHD1 and RNR to Sensitize Cells towards Cytarabine

With the discovery of the ara-CTPase activity of the dNTP triphosphohydrolase SAM and HD domain-containing protein-1 (SAMHD1) resulting in limited ara-C activity in SAMHD1^+^ cancer cells as well as xenograft models, SAMHD1 was proposed as a novel target in AML patients [102,103,104]. The deoxycytidine analog cytarabine (ara-C) in combination with anthracyclines remains the standard of care in AML patients [105]. After cellular uptake, dCK converts ara-C to its active metabolite ara-CTP, which is then incorporated in DNA leading to DNA damage by perturbating DNA synthesis [106]. However, a lack in response followed by relapse and treatment failure is often observed through the unsuccessful accumulation of ara-CTP demonstrating the need for novel strategies in AML treatment in adults and children [105,106]. Several small-molecule SAMHD1 inhibitors were developed in silico and validated in enzymatic assays; however, none of them demonstrated cellular activity [107,108].

As small molecules failed to inhibit SAMHD1 in vitro, a novel approach was required to sensitize AML cancer cells and xenograft models to cytarabine. Successful targeting of SAMHD1 resulting in increased sensitivity of ara-C could be achieved with the simian immunodeficiency virus (SIV) protein Vpx in AML cell and xenograft models as well as in primary AML patient-derived blasts [103,109]. Vpx results in labeling SAMHD1 for proteasomal degradation and reduces SAMHD1 protein levels [110].

The recent discovery of RNR as a regulator of SAMHD1 activity enabled a novel strategy to overcome the limitations of ara-C. SAMHD1 ara-CTPase activity is dependent on dNTP binding to the regulatory site of the enzyme. Upon RNR inhibition, dNTP synthesis is disabled causing an imbalance in dNTP pools and a decrease in SAMHD1 activity. Pyrimidine RNR inhibitors such as hydroxyurea and gemcitabine were identified to improve ara-C efficacy in *SAMHD1* expressing in vitro and in vivo models as well as in primary patient-derived blasts ex vivo. Surprisingly, no synergistic effects of RNR inhibition with purine analogs and ara-C were observed demonstrating the importance of pyrimidine de novo synthesis in the response rates of AML patients to ara-C (Figure 3C,F) [109].

### 5.4. Targeting of CTPS to Potentiate Gemcitabine and Cytarabin DNA Incorporation

The two human CTPS isoforms, CTPS1 and CTPS2 interconvert UTP to the dCTP precursor CTP as the rate-limiting step in pyrimidine de novo synthesis for successful DNA synthesis to maintain cell proliferation (Figure 1A) [111,112]. Already in the 1970s and 80s, CTPS was suggested to be an attractive target in anti-cancer therapy due to its observed increase in activity leading to elevated CTP levels in lymphocytic and non-lymphocytic leukemia, liver as well as renal carcinoma, and in a variety of other cancers [113,114,115]. With the discovery of increased levels of CTPS1 in lymphoblastic as well as other cancer tissues compared to the unchanged levels of CTPS2 in malignant and healthy tissue, targeting CTPS1 has received renewed interest only recently [116,117,118,119]. More specifically, proteomics analysis of triple-negative breast cancer (TNBC) patient samples revealed an increased expression of CTPS1 compared to para-tumor tissue, which is accompanied by a decrease in disease-free and overall survival of TNBC patients with high levels of CTPS1. CTPS1 silencing in TNBC cancer cell lines decreased proliferation, migration, and invasion as well as increased apoptosis. Furthermore, a reduction in tumor growth was observed in TNBC xenografts upon CTPS1 silencing [116]. Consequently, selective targeting of CTPS1 with small molecules could be a promising new anti-cancer strategy.

The first identified CTPS inhibitor cyclopentenyl cytosine (CPEC) showed initially promising anti-tumor activity in human colon carcinoma, pediatric acute lymphocytic leukemia (ALL) as well as in patient-derived pediatric acute non-lymphocytic leukemia (ANLL) cells (Figure 3F) [120,121,122,123]. Furthermore, CTPS inhibition with CPEC caused a decrease in tumor burden in colon carcinoma and leukemia xenograft models [122,123].

Since ara-C and gemcitabine efficacy is strongly dependent on dCK activity and dCTP is a negative feedback regulator for dCK activity, co-targeting of CTPS with CPEC and either gemcitabine or ara-C was suggested to improve DNA incorporation of both pyrimidine analogs upon dCTP synthesis inhibition (Figure 3D) [90,106]. Inhibition of CTPS with CPEC increased ara-C activity followed by apoptosis induction in T lymphoblastic as well as human neuroblastoma cancer cells [124,125]. A similar effect was observed upon pre-treatment with CPEC followed by gemcitabine leading to increased dFdCTP incorporation accompanied by increased cytotoxicity in lymphocytic and myeloid leukemia cells [126].

Even though initial in vitro and in vivo studies targeting CTPS alone or in combination with cytidine analogs have shown promising results, the findings could not be translated into the clinics. Treatment of colon cancer patients with CPEC in a single Phase I study caused severe cardiovascular toxicity demonstrating the need for new selective CTPS inhibitors [127].

Structural binding analysis of co-crystal structures of the newly developed CTPS1 inhibitors R80 and R80 structural analogs revealed specific binding to CTPS1 and all R80 analogs were potent in enzymatic activity assays on recombinant CTPS1. However, their potential as anti-cancer agents targeting CTPS1 alone or in combination with other standard-of-care pyrimidine synthesis inhibitors must be further evaluated in vitro and in vivo [119].

### 5.5. Co-Targeting of Thymidine Synthase and Thymidine Kinases Sensitizes Cancer Cells towards Traditional Anti-Cancer Agents

The activity of both, cytosolic (TK1) and mitochondrial thymidine kinase (TK2), is upregulated by anti-cancer agents targeting thymidine synthase (TS) in the pyrimidine de novo synthesis pathways. Consequently, co-targeting of pyrimidine salvage via TKs and pyrimidine de novo synthesis via TS inhibition in different cancer cell models was suggested to improve the efficacy of traditional anti-cancer agents.

The inhibition of the TK-mediated pyrimidine salvage is currently only possible via siRNA-induced knockdown of the corresponding enzyme due to the lack of TK-specific inhibitors [84,92,128,129]. Knockdown of the mitochondrial thymidine kinase TK2 via siRNA increased the capacity of TS siRNA to sensitize cervical carcinoma cancer cells as well as breast epithelial adenocarcinoma cancer cells towards the active metabolite 5-fluorodeoxyuridine (5FUdR) of the traditional anti-cancer agent 5-FU. Interestingly, siRNA knockdown of the cytosolic thymidine kinase TK1 but not TK2 caused an increased effect of TS siRNA and increased sensitivity of both cell lines towards the TS targeting folate analog pemetrexed (Figure 3E) [129].

As TK1 is the predominant thymidine kinase present in normal proliferating cells as well as cancer cells, the role of mitochondrial cell-cycle independent TK2 in cancer must be further investigated. The potential of TK1 and TK2 as anti-cancer targets in combination with other pyrimidines de novo synthesis inhibitors has only been demonstrated in vitro so that further in vivo experiments are required to fully understand the potential effect of TKs in cancer.

## 6. Conclusions

Targeting nucleotide synthesis and, consequently, DNA synthesis remains the backbone of cancer therapy besides its limitations caused by the ability of cancer cells to adapt to nucleoside salvage pathways to maintain successful DNA replication.

In recent years, pyrimidine salvage gained new attention leading to the development of new inhibitors for already existing key players as well as to the discovery of novel enzymes involved in pyrimidine salvage. Combination therapy is the go-to in current anti-cancer therapy. This review opens a new perspective of combining inhibitors of pyrimidine salvage and de novo synthesis to overcome the limitations of traditionally used anti-cancer agents. We highlight current targets for the development of new inhibitors to improve overall survival and prognosis in cancer patients.

Even though the targeting of key players of pyrimidine salvage with both new and already established inhibitors alone or in combination with pyrimidine de novo synthesis showed promising results in cancer cell models, it must be further evaluated in vivo as well as in patients to uncover its full potential in cancer therapy.

We furthermore hypothesize that targeting of pyrimidine salvage could not just be of advantage in combination with de novo pyrimidine synthesis inhibition but also with other anti-cancer agents targeting different pathways such as cell cycle regulation or purine metabolism in cancer. However, more research must be completed to identify potential co-targeting strategies in cancer.

## Figures and Tables

**Table 1:** Overview of pyrimidine de novo synthesis inhibitors used in cancer therapy.

Drug Name	Mode of Action	Current Use
5-Fluorouracil (Prodrugs: Floxuridine, capecitabine) 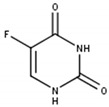	Thymidylate synthase inhibition and RNA synthesis inhibition	Breast cancer Colon cancer Esophageal cancer Stomach cancer Pancreas cancer Head and neck cancer Premalignant skin cancer
Cytarabine (cytosine arabinoside) 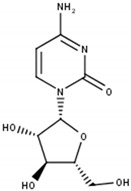	DNA incorporation	Acute myeloid leukemia (AML) Acute lymphocytic leukemia (ALL) Chronic myelogenous leukemia (CML) Lymphoma
Gemcitabine 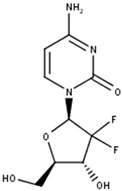	Inhibits ribonucleotide reductase (RNR) and DNA synthesis	Non-small cell lung cancer Gallbladder cancer Bladder cancer Breast cancer Ovarian Cancer Pancreatic cancer

**Table 2:** Overview of a selection of Phase II clinical trials of the CAD inhibitor PALA, the DHODH inhibitor Brequinar, and the UMPS inhibitor Pyrazofurin.

Drug Name and Mode of Action	Clinical Trials	Status	Observations and Side Effects
PALA CAD Inhibition 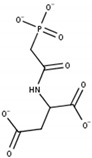	*Kleeberg et al., 1982* Advanced breast cancer	not approved	No response Mucocutaneous toxicity and diarrhea
	*Paridaens et al., 1982* Malignant melanoma		7% complete response Mucocutaneous toxicity and ocular manifestations
Brequinar DHODH inhibition 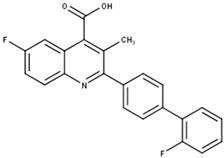	*Dodion et al., 1990* Metastatic colorectal cancer	not approved in cancer FDA-approved for rheumatoid arthritis and multiple sclerosis data	No response Severe toxicity, thrombocytopenia
	*Urba et al., 1992* Advanced squamous-cell carcinoma of the head and neck		No response Moderate toxicity, thrombocytopenia, diarrhea
	*Cody et al., 1993* Advanced breast cancer		12% partial response Moderate toxicity
	*Maroun et al., 1993* Advanced lung cancer		6% partial response Moderate toxicity, thrombocytopenia
	*Moore et al., 1993* Advanced gastrointestinal cancer		3% response in colorectal carcinoma; 7% in gastric carcinoma; no response in pancreatic cancer Moderate toxicity; two treatment-related deaths
Pyrazofurin UMPS inhibition 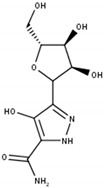	*Creagan et al., 1977* Advanced colorectal carcinoma	not approved	No response Nausea, vomiting, stomatitis
	*Nichols et al., 1978* Advanced breast cancer		No response Moderate to severe stomatitis, thrombocytopenia
	*Carroll et al., 1979* Advanced colorectal carcinoma		No response Normochromic normocytic anemia
